# Measurement properties of the OARSI core set of performance-based measures for hip osteoarthritis: a prospective cohort study on reliability, construct validity and responsiveness in 90 hip osteo­arthritis patients

**DOI:** 10.1080/17453674.2018.1539567

**Published:** 2018-11-19

**Authors:** Jaap J Tolk, Rob P A Janssen, C (Sanna) A C Prinsen, M (Marieke) C van der Steen, Sita M A Bierma Zeinstra, Max Reijman

**Affiliations:** a Department of Orthopedic Surgery and Trauma, Máxima Medical Center, Eindhoven;;; b Department of Epidemiology and Biostatistics, VU University Medical Center, Amsterdam Public Health (APH) Research Institute, Amsterdam;; c Department of Orthopedic Surgery, Catharina Hospital Eindhoven, Eindhoven;;; d Department of General Practice, Erasmus MC, University Medical Center Rotterdam, Rotterdam;;; e Department of Orthopedic Surgery, Erasmus MC, University Medical Center Rotterdam, Rotterdam, The Netherlands

## Abstract

Background and purpose — Improvement of physical function is one of the main treatment goals in severe hip osteoarthritis (OA) patients. The Osteoarthritis Research Society International (OARSI) has identified a core set of performance-based tests to assess the construct physical function: 30-s chair stand test (30-s CST), 4x10-meter fast-paced walk test (40 m FPWT), and a stair-climb test. Despite this recommendation, available evidence on the measurement properties is limited. We evaluated the reliability, validity, and responsiveness of these performance-based measures in patients with hip OA scheduled for total hip arthroplasty (THA).

Patients and methods — Baseline and 12-month follow-up measurements were prospectively obtained in 90 end-stage hip OA patients who underwent THA. As there is no gold standard for comparison, the hypothesis testing method was used for construct validity and responsiveness analysis. A test can be assumed valid if ≥75% of predefined hypotheses are confirmed. A subgroup (n = 30) underwent test–retest measurements for reliability analysis. The Oxford Hip Score, Hip injury and Osteoarthritis Outcome Score—Physical Function Short Form, pain during activity score, and muscle strength were used as comparator instruments.

Results — Test–retest reliability was appropriate; intraclass correlation coefficient values exceeded 0.70 for all 3 tests. None of the performance-based measures reached 75% hypothesis confirmation for the construct validity or responsiveness analysis.

Interpretation — The performance-based tests have good reliability in the assessment of physical function. Construct validity and responsiveness, using patient-reported measures and muscle strength as comparator instruments, could not be confirmed. Therefore, our findings do not justify their use for clinical practice.

Improvement of physical function is one of the main treatment goals of total hip arthroplasty (THA). Physical function can be assessed using patient-reported and performance-based outcome measurement instruments (Reiman and Manske 2011). Because different domains of the construct physical function are measured, the methods are considered complementary and not competing (Stratford and Kennedy 2006, Reiman and Manske 2011, Dobson et al. 2013).

3 activities have been identified as most relevant for patients with hip OA: sit-to-stand movement, level walking, and stair negotiation (Dobson et al. 2013). Impairment on these domains is classified as “activity limitations” on the World Health Organization International Classification of Functioning, Disability and Health (ICF) (World Health Organization 2001). The Osteoarthritis Research Society International (OARSI) has identified a set of performance-based tests to assess the construct physical function (Dobson et al. 2012, 2013). The core set consists of the 30-s chair stand test (30-s CST) for assessment of sit-to-stand movement, 4x10 meter fast-paced walk test (40 m FPWT) for assessment of level walking, and a stair-climb test to assess stair negotiation (Dobson et al. 2013).

The validity and responsiveness of the OARSI core set have been challenged in knee OA patients (Tolk et al. 2017), but available evidence on the measurement properties in patients with hip OA is insufficient (Dobson et al. 2012, 2013). Measurement properties of a test should be confirmed in the population in which it is to be used, but the recommendation to use the specific tests included in the OARSI core set is based on expert opinion (Dobson et al. 2012, 2013). Therefore, before further implementation of the OARSI core set for hip OA patients can be considered, additional evidence on the measurement properties of these performance measures is essential (Terwee et al. 2006, Dobson et al. 2012). We evaluated the reliability, validity, and responsiveness after THA of the OARSI recommended performance-based measures, for measurement of physical function in patients with severe hip OA.

## Patients and methods

We performed a prospective cohort study of patients indicated for THA to evaluate the measurement properties of the 30-s CST, 40 m FPWT, and 10-step stair climb test (10-step SCT). The study was conducted following the COSMIN (COnsensus based Standards for the selection of health status Measurement INstruments) checklist (Mokkink et al. 2010b). The COSMIN checklist contains design requirements and preferred statistical methods for studies on measurement properties of health status measurement instruments.

### Patient population

Patients were eligible for inclusion if they had unilateral symptomatic hip OA and were scheduled for primary THA. Patients with comorbidity leading to inability to perform the performance-based measures, insufficient knowledge of the Dutch language, and inability to visit follow-up appointments were excluded. All patients in the Máxima Medical Centre meeting these criteria, and willing to participate, signed an informed consent form. The number of patients needed for the analysis was guided by the COSMIN standards (Terwee et al. 2007, Mokkink et al. 2010b). We aimed to include ≥50 patients for construct validity and responsiveness analyses, and 30 patients for reliability analyses.

### Study procedures

Patient characteristics measured at baseline were: sex, age, and BMI. The assessment of performance-based measures and comparator instruments described below was made at baseline before surgery, and 12 months after THA. The standardized testing procedures were performed by a research nurse strictly according to the manual provided by the OARSI, with a fixed order of tests (Dobson et al. 2013).

### Performance-based measures

***30-s CST.*** The 30-s CST aims to quantify a patient’s performance on the activity “sit-to-stand movement” (Dobson et al. 2013). From a sitting position, the patient stands up until hips and knees are fully extended, then completely back down. This is repeated for 30 seconds and each full cycle is counted as 1 chair stand (Dobson et al. 2013). A 43-cm high, straight-back chair without armrests was used. For patients with hip OA, good reliability is reported with an intraclass correlation coefficient (ICC) of 0.81 (0.63–0.91) and standard error of measurement (SEM) of 1.27 (Wright et al. 2011). No reports on construct validity are available.

***40 m FPWT.*** The 40 m FPWT is a test for performance on the activity short-distance walking (Dobson et al. 2013). Participants are asked to walk as quickly but as safely as possible, without running, along a 10-meter walkway for a total distance of 40 meters. Walking speed is measured in meters/second (m/s). Use of a walking aid is allowed and recorded. Inter-rater reliability is reported to be good in patients with hip OA, with an ICC of 0.95 (0.90–0.98) and SEM of 1.0 m/s (Wright et al. 2011). There are no reports available on the construct validity.

***Stair climb test.*** The OARSI included a stair-climb test in the core set, but no specific measure is recommended (Dobson et al. 2013). We selected the 10-step stair climb test (10-step SCT), as the stair in the testing area had 10 steps with a step height of 19 cm. Patients were instructed to ascend and descend the flight of stairs as quickly as possible but in a safe manner. The time needed is recorded in seconds (Dobson et al. 2013). To our knowledge, there is no evidence available on measurement properties of the 10-step stair-climb test or comparable stair-climb tests in patients with hip OA.

#### Comparator instruments

We used a combination of comparator instruments; a specification of these instruments and their measurement properties can be found in a supplementary file. For measurement of physical function 2 joint-specific PROMs were used: the Hip injury and Osteoarthritis Outcome Score—Physical Function Short Form (HOOS—PS) (Davis et al. 2009), and the Oxford Hip Score (OHS) (Dawson et al. 1996). The EuroQol 5D-3L (EQ-5D) was used as a measure of health-related quality of life (Rabin and de Charro 2001). Pain during activity was scored from 0 to 10 using a numerical rating scale (NRS pain) (Ruyssen-Witrand et al. 2011). At 12 months follow-up a 7-point Likert scale anchor question was scored for change in activities of daily living. Preoperatively knee extensor and hip abductor strength of the affected leg was measured using a handheld dynamometer (Holstege et al. 2011, Zeni et al. 2014).

### Evaluation of the measurement properties and statistics

#### Reliability

Test–retest reliability refers to the extent to which scores for patients who have not changed are the same for repeated measurement over time. For this analysis, test–retest measurements of the 3 performance-based measures were obtained in a subset of the study population. 30 minutes of rest were allowed in between, to allow for full recovery during the resting interval. Performance on the activity under study can assumed to be stable over this testing period. ICC values for absolute agreement with corresponding 95% confidence intervals (CI) were calculated using a 2-way random model with absolute agreement. The threshold for an appropriate ICC is 0.70 (Terwee et al. 2007, Prinsen et al. 2016). SEM and SDC were calculated as described by Atkinson (1998).

#### Construct validity

Construct validity refers to the degree to which the instruments under study measure the construct they aim to measure. This is the recommended method to assess validity when there is no “Gold Standard” available, as is the case for the functional domains level walking, stair negotiation, and sit-to-stand movement in hip OA. Before the start of the study, an expert panel formulated hypotheses on the expected relationships of performance-based measure scores with scores on the comparative instruments (Table 3, see Supplementary data) (Mokkink et al. 2010a, de Vet et al. 2011). Direction and magnitude of the expected results were stated. The expert panel consisted of an orthopedic surgeon (RJ), orthopedic resident and PhD candidate (JT), specialist in measurement property analysis (CP), and a methodologist (MR).

The hypotheses were based on the following predictions—we expected: a moderate correlation of the performance-based measures with PROMs and quadriceps strength; a stronger correlation of PROMs with pain scores than with the performance-based measures; a stronger correlation of the performance-based measures with PROMs measuring functional outcome than with a PROM measuring general health; a stronger correlation of specific questions of the PROMs regarding walking, stair negotiation, and sit-to-stand movement to their respective performance-based measure than to the total PROM score. Correlations on a convergent hypothesis were expected to be at least moderate: ≥ 0.4 or ≤ –0.4. Divergent hypotheses were expected to have a poor correlation (≥ –0.39; ≤ 0.39). Pearson or Spearman correlation coefficients were calculated, depending on normality of data distribution. Construct validity can be assumed adequate if at least 75% of the predefined hypotheses are confirmed (Terwee et al. 2007).

#### Responsiveness

Responsiveness refers to the ability of the instruments to detect change over time in the construct measured. In the absence of a gold standard, a construct approach is to be used. Hypotheses were formulated a priori by the expert panel, in a similar manner to the construct validity analysis (Table 5) (Terwee et al. 2007, Mokkink et al. 2010a, de Vet et al. 2011).

**Table 1.** Patient characteristics. Data are mean (SD) unless otherwise stated

	Total cohort	Reliability analysis
	(n = 90)	cohort (n = 30)
Age, years	69 (9.5)	66 (9.4)
Women, n	61	22
BMI	27 (3.9)	26 (2.7)
Hip abductor strength, N	196 (7.8)	219 (7.9)
Knee extensor strength, N	134 (5.7)	13 (4.3)

**Table 5.** Responsiveness

Predefined hypotheses	3a0-s chair stand test (change score)		40 m fast-paced walk test (change score)		10-step stair climb test	
Spearman correlation coefficient	Hypothesis confirmed	(change score)	Spearman correlation coefficient	Hypothesis confirmed	Spearman correlation coefficient**^a^**	Hypothesis confirmed
1. Moderate correlation with anchor question (≥ 0.4)	0.37	No	0.28	No	–0.18	No
2. Moderate correlation with change score NRS pain during activity (≤ –0.4)	–0.04	No	–0.13	No	0.14	No
3. Moderate correlation with change score HOOS-PS (≤ –0.4)	0.30	No	0.21	No	–0.35	No
4. Moderate correlation with change OHS (≥ 0.4)	0.23	No	0.27	No	–0.26	No
5. Correlation between change scores NRS pain and HOOS-PS is minimal 0.1 stronger than between NRS pain and performance-based test	–0.45/–0.04	Yes	–0.45/–0.13	Yes	–0.45/–0.18	Yes
6. Correlation between change scores NRS pain and HOOS-PS is minimal 0.1 stronger than between HOOS-PS and performance-based test	–0.45/0.30	Yes	–0.45/0.21	Yes	–0.45/–0.35	Yes
7. Correlation between changes scores NRS pain and OHS minimal 0.1 stronger than between NRS pain and performance-based test	–0.66/–0.04	Yes	–0.66/–0.13	Yes	–0.66/–0.18	Yes
8. Correlation between change scores NRS pain and OHS is minimal 0.1 stronger than between OHS and performance-based test	–0.66/0.23	Yes	–0.66/0.27	Yes	–0.66/–0.26	Yes
Hypothesis confirmed	4/8		4/8		4/8	

The hypotheses were formulated according to the following criteria: the anchor question would be moderately correlated to change in the performance-based measures scores (≥ 0.4 or ≤ –0.4) and the change in PROMs would be more correlated to pain than to change in the performance-based measure scores. Pearson or Spearman correlation coefficients were calculated, depending on normality of data distribution. Adequate responsiveness can be assumed if minimally 75% of the predefined hypotheses are confirmed (Terwee et al. 2007).

SPSS statistics version 24.0 was used for the analyses (IBM Corp, Armonk, NY, USA).

### Ethics, funding, and potential conflicts of interest

The Máxima Medical Centre Medical Ethics Committee approved the study (registration code 2014-73). No funding was received for the present study. The authors declare that there are no conflicts of interest related to this article.

## Results

### Patient characteristics

In the period April to October 2015, 90 consecutive patients scheduled for arthroplasty because of hip OA were recruited (Table 1, Figure).

**Figure:**
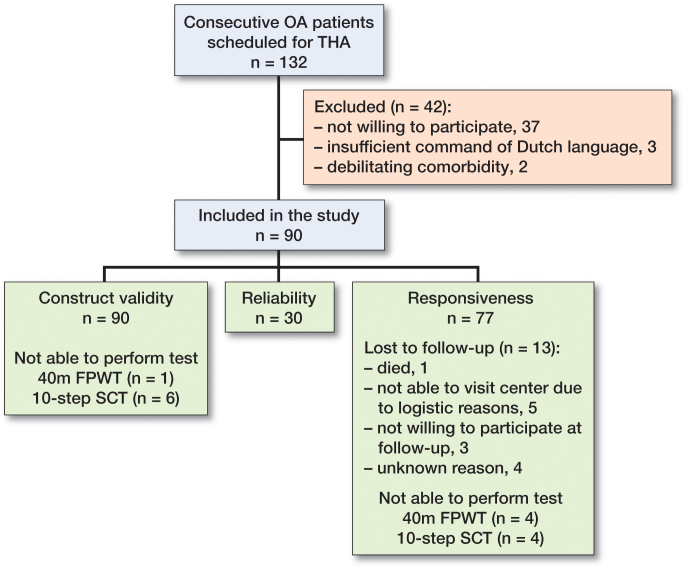
Patients included in the analyses and lost to follow-up.

### Measurement properties

#### Reliability analysis

30 randomly selected patients were enrolled in the test–retest study. Test–retest reliability was appropriate; ICC values exceeded 0.70 for all 3 tests (Table 2, see Supplementary data).

#### Construct validity (hypothesis testing)

None of the 3 performance-based measures reached confirmation of 75% or more of the predefined hypotheses. 4/9 were confirmed for the 30-s CST, 6/17 for the 40m FPWT, and 6/17 for the 10-step SCT (Table 3, see Supplementary data).

#### Responsiveness

The mean score on the anchor question for change in activities of daily living (7-point Likert scale) at 12-month follow-up was 6.2 (5.9–6.4), which represents “much improvement.” Results of the responsiveness analysis are presented in Table 5. For the 30-s CST, 4/8 of the hypothesis were confirmed, for the 40m FPWT 4/8, and for the 10-step SCT 4/8 (Table 4, see Supplementary data).

## Discussion

To our knowledge, this is the first thorough assessment of the measurement properties of the OARSI-recommended core set of performance-based measures in patients with severe hip OA. The reliability analysis showed excellent test–retest reliability, which is in line with previous reports (Wright et al. 2011, Dobson et al. 2017). Construct validity and responsiveness could not be confirmed. These findings are in accordance with recently published work on the OARSI core set of performance-based measures in knee OA patients (Tolk et al. 2017).

All 3 performance-based measures scored poorly on the construct validity and responsiveness analysis. One of the reasons is that almost all convergent hypotheses with PROMs measuring physical function were rejected. Although both methods aim to quantify related constructs, previous research has shown that PROMs assessing physical function do not measure the exact same domain as performance-based measures (Stratford and Kennedy 2006, Reiman and Manske 2011, Dobson et al. 2013). This potentially limits the strength of the conclusions that can be drawn from the present study. For example, PROMs are known to have a higher dependency on pain scores than performance based-measures (Stratford and Kennedy 2006). When—in the absence of a gold standard—the construct approach is to be used, it is inherently so that there is a discrepancy between the test under study and the comparator instruments (de Vet et al. 2011). Furthermore, PROMs were not the only comparative instruments used, and hypotheses predicting a higher correlation of the performance-based measure scores with related construct compared with less related constructs were largely rejected as well. Therefore, in our opinion, the conclusion on the construct validity and responsiveness should be interpreted more broadly than only showing the known discrepancy between PROMs and these measures.

As an alternative to the comparator instruments used for construct validity and responsiveness in the present study, 3-D motion analysis or inertia-based motion analysis could be used. These methods allow for a kinematic analysis in patients with hip OA, but their clinical relevance has not been defined (Kolk et al. 2014, Bolink et al. 2016). Therefore, we believe these alternative methods are not suitable for comparison purposes in a clinical perspective. The comparative instruments used in the present study were considered the most suitable instruments available.

The findings on construct validity of the performance-based measures might be affected because impairment on the tested activities in daily living is not fully appreciated by merely timing the performance (Steultjens et al. 1999, Stratford and Kennedy 2006). Although others claim good face validity for the core set of performance-based measures (Dobson et al. 2013, 2017), in our view this is not straightforward. For example, standing up and sitting down in rapid sequence, as measured by the 30-s CST, is not really exemplary for stand-to-sit movement in daily life. Fewer repetitions on the test does not necessarily mean the quality of a sit-to-stand movement in daily living is more or less impaired. The same goes for walking speed and stair ascent, which does not directly represent more or less impairment. Merely timing the activity or counting repetitions cannot capture impairment caused by limping or joint instability, nor avoidance of an activity in daily living (Steultjens et al. 1999, Holla et al. 2014). This is a possible explanation as to why the construct validity could not be confirmed.

The responsiveness analysis showed that change in pain scores was strongly correlated to change in PROM scores, but not related to performance-based measure scores. Others have presented this low correlation with pain scores as a strength of performance-based measures, claiming this makes them more “objective” (Dobson et al. 2012, 2013). In our opinion, it seems unlikely that the degree of pain during an activity would not influence performance in daily living (Holla et al. 2014). Furthermore, it has been shown that pain during activity does affect the quality of movement, and impaired quality of movement is associated with lower perceived physical function (Steultjens et al. 1999, Rosenlund et al. 2016). Although pain reduction is not related to an increase in speed on the tested activities, the quality and manner of performance might improve (Steultjens et al. 1999), and patients might no longer avoid the activities (Holla et al. 2014). These factors of physical performance are not grasped by the performance-based measures under study. The number of repetitions or speed scored on the performance-based measures might be of interest for research purposes, but tin the authors’ opinion actual change and perceived change need to be related to some degree for a test to be clinically relevant. Hypotheses in this regard were all rejected, contributing to the negative conclusion on the responsiveness of the OARSI core set of performance-based measures.

The strict adherence to the methodological criteria provided by COSMIN is a strength of the present study (Mokkink et al. 2010b). Most previous reports on the measurement properties of the performance-based measures under study reported combined groups of hip and knee OA patients, resulting in heterogeneous populations (Kennedy et al. 2005, Gill and McBurney 2008, Dobson et al. 2017). The present study reports on an unselected, consecutive group of only end-stage hip OA patients. The results can therefore be considered more accurate and representative for this population.

The group size for test–retest measurements was kept relatively small, to reduce the burden of repeated measurements for patients. As there is evidence from other studies showing similar results on reliability (Kennedy et al. 2005, Wright et al. 2011, Dobson et al. 2017), in our view it can be concluded that the performance-based measures under study have adequate test–retest reliability. The percentage of patients lost to follow-up for the responsiveness analysis was 14%. In our opinion, this can be considered acceptable, especially as the group of patients with incomplete data did not show systematic difference in baseline characteristics (Table 1).

In summary, the 30-s CST, 40 m FPWT, and 10-step SCT have good reliability in the assessment of the domains sit-to-stand movement, walking short distances, and stair negotiation in the construct physical function. Construct validity and responsiveness, using patient-reported measures and muscle strength as comparator instruments, could not be confirmed. Therefore, the present study does not justify their use for clinical practice in patients with severe hip OA.

### Supplementary data

Tables 2–4 and a specification of comparator instruments used are available as supplementary data in the online version of this article, http://dx.doi.org/10.1080/17453674.2018.1539567

JT and MR contributed to the conception and design of the study and drafting of the article. CP provided methodological support. All authors contributed to interpretation of the data and critically revised the article.

The authors would like to sincerely thank C. van Doesburg, H. Kox, D. Latijnhouwers, and M. Mariam for their work in administrative and testing procedures.

*Acta* thanks Margareta Hedstrom and Anders Holsgaard-Larsenfor help with peer review of this study.

## Supplementary Material

Supplemental Material
