# Supplementary material for: Measurement properties of the OARSI core set of performance-based measures for hip osteoarthritis: a prospective cohort study on reliability, construct validity and responsiveness in 90 hip osteo­arthritis patients
Source: Acta Orthop. 2018 Nov 19;90(1):15–20. doi: 10.1080/17453674.2018.1539567 (PMC6366472; doi:10.1080/17453674.2018.1539567)
Supplement: Supplemental Material [file IORT_A_1539567_SM7598.pdf]

Supplementary data

Supplementary file – specification of comparator instruments used

Comparator instruments

HOOS-PS

The Hip injury and Osteoarthritis Outcome Score - Physical Function Short Form (HOOS-PS) is a 5-item PROM for measurement of the construct physical function. The HOOS-PS is scored on a 0 to 100 scale, 0 indicating no symptoms and 100 indicating extreme symptoms (Davis et al. 2009). The HOOS-PS has good construct validity and responsiveness in hip OA patients (Davis et al. 2009).

OHS

The Oxford Hip Score (OHS) is a 12-item disease specific PROM for measurement of pain and function of the hip in relation to different activities of daily life. The total score ranges from 12 indicating no difficulties symptoms to 60 indicating most difficulties (Dawson et al. 1996). The OHS has shown to be consistent, reliable, valid and sensitive to clinical change (Dawson et al. 1996, Gosens et al. 2005).

EQ-5D

EuroQol 5D-3L (EQ-5D) is a standardized instrument developed as a measure of health-related quality of life (Rabin and de Charro 2001). This PROM consists of a 5-question descriptive part and a visual analogue scale score (EQ-VAS) ranging from 0 to 100 (Rabin and de Charro 2001). From the 5-question part a sum score can be calculated, where 1 represents the best possible health state and lower scores represent worse health state (Rabin and de Charro 2001). The EQ-5D has shown to be valid and reliable in hip OA patients (Conner-Spady et al. 2015).

NRS pain

Pain during activity was scores using a numerical rating scale (NRS pain). Patients were asked to score pain during activity in the past week on an 11-point scale, the patients rate their pain during activity from 0 to 10. A score of 0 represented 'no pain' and a score of 10 represented 'worst imaginable pain'. Good reliability and responsiveness are reported for this NRS pain scale (Ruyssen-Witrand et al. 2011).

Anchor question

At 12-months follow-up a 7-point Likert scale anchor question was scored for change in activities of daily living. The question 'how has your general daily functioning changed since the operation on your knee?' was scored from 1 (a lot worse) to 7 (very much improved).

Muscle strength

Strength of the knee extensors and hip abductors of the affected leg were tested for all subjects in the study. Maximal isometric knee extensor strength was measured in Newton (N) using a handheld dynamometer (HHD). In an upright sitting position, the HHD was positioned on the anterior aspect of the tibia, five cm proximal to the medial malleolus. A protective shin guard was used for patient comfort as well as standardization of HHD placement. Hip abductor strength was measured with subjects in supine position and with 5° of hip abduction. The HHD was positioned on the lateral femoral condyle and its position was held constant between trials to avoid changes in the resistance moment arm. For both muscle groups three consecutive measurements were obtained, the highest value was used for analysis. The HHD is a widely used instrument to measure knee extensor and hip abductor strength, with good reliability in OA patients. An ICC of 0.94–0.97 is reported (Holstege et al. 2011, Zeni et al. 2014).

Conner-Spady B L, Marshall D A, Bohm E, Dunbar M J, Loucks L, Khudairy A Al, Noseworthy T W. Reliability and validity of the EQ-5D-5L compared to the EQ-5D-3L in patients with osteoarthritis referred for hip and knee replacement. Qual Life Res 2015; 24(7): 1775-84.

Davis A M, Perruccio A V, Canizares M, Hawker G A, Roos E M, Maillefert J-F, Lohmander L S. Comparative, validity and responsiveness of the HOOS-PS and KOOS-PS to the WOMAC physical function subscale in total joint replacement for osteoarthritis. Osteoarthritis Cartilage 2009; 17(7): 843-7.

Dawson J, Fitzpatrick R, Carr A, Murray D. Questionnaire on the perceptions of patients about total hip replacement. J Bone Joint Surg Br 1996; 78(2): 185-90.

Gosens T, Hoefnagels N, de Vet R, Dhert W, van Langelaan E, Bulstra S, Geesink R. The 'Oxford Heup Score': the translation and validation of a questionnaire into Dutch to evaluate the results of total hip arthroplasty. Acta Orthop 2005; 76(2): 204-11.

Holstege M S, Lindeboom R, Lucas C. Preoperative quadriceps strength as a predictor for short-term functional outcome after total hip replacement. Arch Phys Med Rehabil 2011; 92(2): 236-41.

Rabin R, de Charro F. EQ-5D: a measure of health status from the EuroQol Group. Ann Med 2001; 33(5): 337-43.

Ruyssen-Witrand A, Fernandez-Lopez C J, Gossec L, Anract P, Courpied J P, Dougados M. Psychometric properties of the OARSI/OMERACT osteoarthritis pain and functional impairment scales: ICOAP, KOOS-PS and HOOS-PS. Clin Exp Rheumatol 2011; 29(2): 231-7.

Zeni J, Abujaber S, Pozzi F, Raisis L. Relationship between strength, pain, and different measures of functional ability in patients with end-stage hip osteoarthritis. Arthritis Care Res (Hoboken) 2014; 66(10): 1506–12.

Table 2. Reliability analysis (n = 30)

	Score baseline mean (95% CI)	Retest score mean (95% CI)	Difference baseline–retest score mean (95% CI)	ICC (95% CI)	SEM	SDC
30-s CST (stands)	10.1 (9.0–11.2)	10.9 (9.7–12.1)	–0.8 (–0.3 to –1.4)	0.86 (0.66–0.94)	0.99	2.7
40 m FPWT (m/s)	1.32 (1.22–1.43)	1.33 (1.20–1.46)	–0.01 (–0.05 to 0.04)	0.94 (0.88–0.97)	0.08	0.22
10-step SCT (s)	14.2 (12.3–16.0)	14.1 (12.3–15.9)	–0.1 (–0.5 to 0.6)	0.96 (0.91–0.98)	1.06	2.9

ICC, intraclass correlation coefficient; SEM, standard error of measurement; SDC, smallest detectable change.

Table 3. Construct validity

Predefined hypotheses	30-s chair stand test Spearman correlation coefficient	30-s chair stand test Hypothesis confirmed	40 m fast-paced walk test Spearman correlation coefficient	40 m fast-paced walk test Hypothesis confirmed	10-step stair climb test Spearman correlation coefficient [a]	10-step stair climb test Hypothesis confirmed
1. Moderate correlation with HOOS-PS (≤ –0.4) *	0.21	No	0.21	No	–0.24	No
2. Moderate correlation with OHS (≥ 0.4) *	0.45	Yes	0.34	No	–0.27	No
3. Moderate correlation with hip abductor strength (≥ 0.4) *	0.21	No	0.48	Yes	–0.44	Yes
4. Moderate correlation with quadriceps strength (≥ 0.4) *	0.35	No	0.46	Yes	–0.53	Yes
5. Unrelated to EQ-5D (–0.39; 0.39)	0.38	Yes	0.31	Yes	0.34	Yes
6. Correlation with HOOS-PS is minimal 0.1 stronger than with EQ-5D	0.21/0.38	No	0.21/0.31	No	–0.24/0.34	No
7. Correlation with OHS is minimal 0.1 stronger than with EQ-5D	0.45/0.38	No	0.34/0.21	Yes	–0.27/0.34	No
8. "Absolute" correlation between NRS pain and HOOS-PS is minimal 0.1 higher than between performance-based measure and NRS pain	–0.53/–0.19	Yes	–0.53/–0.12	Yes	–0.53/0.02	Yes
9. "Absolute" correlation between NRS pain and OHS is minimal 0.1 higher than perfor-mance-based measure and NRS pain	–0.63/–0.19	Yes	–0.63/–0.12	Yes	–0.63/0.02	Yes
10. "Absolute" correlation 40 m FPWT with HOOS-PS Question 4 is minimal 0.1 stronger than with HOOS-PS	NA		–0.12/0.21	No	NA	
11. "Absolute" 40 m FPWT with HOOS-PS Question 4 is minimal 0.1 stronger than with OHS	NA		–0.12/0.34	No	NA	
12. "Absolute" correlation 40 m FPWT with HOOS-PS Question 4 is minimal 0.1 higher than with EQ-5D Score	NA		–0.12/0.31	No	NA	
13. "Absolute" correlation 40 m FPWT with OHS Question 4 is minimal 0.1 stronger than with HOOS-PS	NA		–0.12/0.21	No	NA	
14. "Absolute" correlation 40 m FPWT with OHS Question 4 is minimal 0.1 stronger than with OHS	NA		–0.12/0.34	No	NA	
15. "Absolute" correlation 40 m FPWT with OHS Question 4 is minimal 0.1 stronger than with EQ-5D Score	NA		–0.12/0.31	No	NA	
16. Moderate correlation 40 m FPWT with EQ-5D Question 1 (≤ –0.4)	NA		–0.36	No	NA	
17. Moderate correlation 40 m FPWT with OHS Question 4 (≤ –0.4)	NA		–0.12	No	NA	
18. "Absolute" correlation 10-step SCT with OHS Question 6 is minimal 0.1 stronger than with HOOS-PS	NA		NA		0.31/–0.24	No
19. "Absolute" correlation 10-step SCT with OHS Question 6 is minimal 0.1 stronger than with OHS	NA		NA		0.31/–0.27	No
20. "Absolute" correlation 10-step SCT with OHS Question 6 is minimal 0.1 stronger than with EQ-5D	NA		NA		0.31/–0.31	No
21. Moderate correlation 10-step SCT with OHS Question 6 (≤ –0.4)	NA		NA		0.31	No
22. "Absolute" correlation 10-step SCT with HOOS-PS Question 1 is minimal 0.1 stronger than with HOOS-PS	NA		NA		0.34/–0.24	Yes
23. "Absolute" correlation 10-step SCT with HOOS-PS question 1 is minimal 0.1 stronger than with OHS	NA		NA		0.34/–0.27	No
24. "Absolute" correlation 10-step SCT with HOOS-PS question 1 is minimal 0.1 stronger than with EQ-5D	NA		NA		0.34/–0.31	No
25. Moderate correlation 10-step SCT with HOOS-PS question 1 (≤ –0.4)	NA		NA		0.34	No
Hypothesis confirmed	4/9		6/17		6/17	

NA = not applicable.
[a] The 10-step SCT is scored in the opposite direction of the 30-s CST and 40 m FPWT (better performance is a lower score) therefore the hypothesized correlations are in the opposite direction.

Table 4. Performance-based measures and PROM scores before and after THA. Data are mean (SD) unless otherwise stated

Item	Baseline	12-month follow-up	p-value
30-s CST (stands)	9.3 (8.5–10.2)	12.0 (11.2–12.9)	< 0.001
40 m FPWT (m/s)	1.26 (1.17–1.34)	1.34 (1.26–1.42)	< 0.001
Use of assistive device during			
40m FPWT (patients, n)	8	2	0.057
10-step SCT (seconds)	17.9 (15.3–20.4)	14.5 (12.9–16.2)	< 0.001
Use of handrail during			
10-step SCT (patients, n)	41	28	0.047
HOOS-PS score	48.0 (44.3–51.9)	21.7 (19.8–26.2)	< 0.001
OHS	23.6 (21.9–25.7)	41.8 (40.5–43.2)	< 0.001
EQ-5D	0.51 (0.43–0.57)	0.83 (0.79–0.86)	< 0.001
EQ-VAS	64.8 (59.6–70.0)	76.1 (71.5–80.7)	0.001
NRS pain	6.8 (6.5–7.3)	1.5 (1.7)	< 0.001
Anchor question (patients, n)			
Very much improvement		34	
Much improvement		33	
A little improvement		5	
Unchanged		1	
A little worse		0	
Much worse		4	
Very much worse		0	
